# Preventive role of community-level social capital in the need for long-term care and impairment in instrumental activities of daily living: a multilevel analysis

**DOI:** 10.1265/ehpm.22-00126

**Published:** 2023-02-07

**Authors:** Hitomi Matsuura, Yoko Hatono, Isao Saito

**Affiliations:** 1 Health and Welfare Division, Ehime Prefectural Office, Japan; 2 Department of Health Sciences, Faculty of Medical Sciences, Kyushu University, Fukuoka, Japan; 3 Department of Public Health and Epidemiology, Faculty of Medicine, Oita University, Yufu, Japan; 4 Department of Health Sciences, Graduate School of Medical Sciences, Kyushu University, Fukuoka, Japan

**Keywords:** Social capital, Reciprocity, Social cohesion, Need for long-term care, Multilevel analysis

## Abstract

**Background:**

Individual-level social capital is an important determinant of older adults’ long-term care needs; however, there is scant evidence regarding community-level social capital. Therefore, we investigated the association between community-level social capital and the prevalence of the need for long-term care among older adults.

**Methods:**

Between January and February 2018, a cross-sectional survey was conducted among all older adults (n = 13,558) aged 65 to 74 years in a rural municipality in Japan (total population, n = 72,833). A self-reported questionnaire was used to identify community-level social capital, comprising civic participation, social cohesion, and reciprocity. A multilevel logistic regression analysis was performed to estimate the odds ratios of the need for long-term care and a decline in social activity competence as assessed by instrumental activities of daily living. For the analysis, the community levels were divided into 76 voting districts and adjusted for daily life, lifestyle, socioeconomic status, health conditions, and the three social capital subscale scores at the individual level.

**Results:**

After adjusting for the covariates, we observed a tendency that a higher community level of reciprocity was associated with a lower prevalence of long-term care needs (OR: 0.86, 95% confidence interval: 0.75–1.00), whereas a high community level of social cohesion was associated with a significantly reduced decline in instrumental activities of daily living (OR per standard deviation increase: 0.87, 95% confidence interval: 0.79–0.96). No significant association was found with civic participation. Similarly, individual-level social capital was associated with the need for long-term care and decline in instrumental activities of daily living.

**Conclusions:**

Our findings suggest that good community-level reciprocity or social cohesion as well as good individual social capital status may help prevent the need for long-term care among older adults.

**Supplementary information:**

The online version contains supplementary material available at https://doi.org/10.1265/ehpm.22-00126.

## Background

The population aging rate in Japan, a super-aging society, reached 28.4% in 2019. [1] Extending healthy life expectancy, including living prolonged elderly years in good health, is important not only for improving the quality of life of individuals and their families, but also for reducing medical expenses and long-term care (LTC) in an entire society. Therefore, to decrease LTC-related burden, it is important to prevent the need for LTC. [2]

Under such circumstances, social capital has garnered attention as a concept that describes creating a community built on residents’ initiatives and mutual assistance. Putnam [3] described social capital by referring to the “features of social organization, such as trust, norms, and networks, that could improve the efficiency of society by facilitating coordinated actions.” The accumulated evidence on social capital suggests that it is an important social factor in personal health. [4] However, in Japan and worldwide, there is little evidence concerning the contribution of community social capital to prevent the need for LTC.

Individual-level social capital, which includes interactions with family and friends, along with the mutual aid that fosters them, effectively prevents the need for LTC. [5, 6] Several ecological studies considering the rate of participation in hobbies or local groups as a social capital indicator in municipal units indicated that regions with high community-level social capital had lower rates of recognized nursing care needs. [7–9] A Japanese cohort study reported that community-dwelling women with high mistrust had a disability onset rate of 1.68 times higher than those without, even after adjusting for covariates. [10] Furthermore, social cohesion reduced the risk of the onset of functional disability (hazard ratio 0.910) among men after adjusting for individual social and behavioral variables. [11] The Japan Gerontological Evaluation Study used data from each municipality’s school district area. For older adults, however, many studies have revealed a positive impact of neighborhoods on activity levels. [12] Therefore, using community levels, defined as small daily living areas, where older adults could be influenced by their neighbors, a multilevel analysis is necessary to examine the impact of social capital on the need for LTC. [13] However, few multilevel studies have assessed the association between social capital and the prevalence of the need for LTC.

Instrumental activities of daily living (IADL) represent older adults’ ability to perform activities related to independent living [14–18] in domains such as public transportation and shopping for daily necessities, the precursor of the need for LTC. [19–21] Studies on the association between social capital and IADL have not yielded consistent results at the community level. [22, 23] We hypothesized that older adults living in areas with high rates of social capital obtain favorable health information, which may lead to maintenance of IADL, thereby preventing the need for LTC.

Therefore, we used voting districts, which are smaller than school districts in Japan, to evaluate the relationship of community-level social capital with IADL and the need for LTC.

## Methods

### Study population

This cross-sectional survey that utilized self-report questionnaires was conducted among all elderly individuals (n = 13,558) aged 65 to 74 years in Uwajima City (72,833 as of April 2021; 39.3% of the population was aged 65 years or above), Ehime Prefecture, Japan. This city was established in 2005 after merging one city and three towns and now includes 76 voting districts. The voting districts vary and are established in mountainous, coastal, and urban areas.

Participants were 13,558 men and women aged between 65 and 75 years (as of December 2017) across all 76 voting districts. A self-administered questionnaire survey was conducted by mail between January and February 2018. Additional file 1 presents the selection process of the participants.

We analyzed data of participants who provided written informed consent by checking the questionnaire collection rate for each voting district, excluding voting districts with a questionnaire collection rate of <25%, and polling districts with fewer than 10 respondents as these data were considered to be a poor representation of the voting district (see Additional file 2). A previous study revealed that in a logistic analysis, events per variable values of 10 or greater indicate no hindrance. [24]

The Ministry of Internal Affairs and Communications requires polling places to be located within 3 km of each resident’s house. Thus, daily activities can be performed on foot. Uwajima City and many other municipalities have implemented this standard in their ordinances.

Valid responses among the participants were those for which they provided data on voting districts, gender, age, long-term care insurance (LTCI) certification status, and social capital indicators. Respondents missing those data were excluded. Consequently, the data of 5,964 individuals from 65 voting districts were included in the analysis. The required sample size was calculated to be 759 (α = 0.05, β = 0.8, effect size = 0.02); thus, the sample size in this study met the requirement. [25]

This study was approved by the Kyushu University Medical Division Ethics Review Board (approval number 29-371) and conformed to the principles of the Declaration of Helsinki.

### Questionnaire content

#### Basic attributes

Basic attributes included gender, age, place of residence, academic background, economic status (ES), and personal medical history. ES is an important predictor of LTC prevention. [26] Academic background was divided into four categories based on the highest level of education completed: compulsory education, high school, university, and others. ES was subjectively classified into one of four levels ranging from comfortable to poor. For personal medical history, we recorded the presence or absence of the following eight conditions: high blood pressure, diabetes, stroke, myocardial infarction, liver disease, kidney disease, cancer, and others. These specific conditions were selected as they have been considered risk factors for LTC needs. [27]

#### LTCI

The presence of LTCI certification was assessed, and individuals with such certification were also asked to indicate the level of care required. In the Japanese LTCI, care eligibility is determined at two support levels and five care need levels based on the severity of physical and cognitive disabilities. Having the “need for support” refers to having a condition that still allows the individual to perform daily activities by oneself but with the need for some support, while having the “need for LTC” refers to having a condition that makes it difficult for the individual to perform daily activities by oneself, thus, requiring LTC. [28] We defined the onset of the requirement for LTC as the point at which a participant was certified as having a care need level of 2 or greater. This level was selected because there is a clear boundary in disability severity between levels 1 and 2: people at level 1 or lower experience instability in rising and gait and require partial support in toileting, bathing, and so on, whereas those at level 2 or greater are completely unable to rise and walk and require partial or complete support for toileting, bathing, and so on.

#### IADL

The Tokyo Metropolitan Institute of Gerontology Index of Competence, which is tailored to the Japanese lifestyle and based on the domains used by Lawton, [14] was used to measure IADL. The 13-item Tokyo Metropolitan Institute of Gerontology Index of Competence measures higher-order social activity competence not captured by activities of daily living measurements. [16] The reliability and validity of this scale have been confirmed (Cronbach’s alpha: 0.913), and they are widely used in Japan. [16] Items include (i) using public transportation; (ii) shopping for daily necessities; (iii) preparing meals; (iv) paying bills; (v) managing deposits at a bank or post office; (vi) completing documents (e.g., pensions); (vii) reading newspapers; (viii) reading books and magazines; (ix) expressing interest in health articles and programs; (x) visiting friends’ homes; (xi) advising family and friends; (xii) visiting someone in the hospital; and (xiii) assertively initiating a conversation with young people. Questions had “yes” or “no” responses, with 1 point added for the former. Total scores ranged from 0 to 13, with higher scores indicating a greater higher-level functional capacity. The cut-off value was set at 10 points; scores of 10 or below indicated a decline in IADL and, therefore, a higher risk for LTC. [29, 30] In this study, TMIG-IC of 10 points or less was defined as IADL decline.

#### Social capital

Social capital indicators were assessed using the regional health-related social capital indicators (ver. 2.0). [31] These social capital indicators were developed in Japan, and their reliability and validity have been confirmed elsewhere (Cronbach’s alpha: 0.752). [31] The scale, which includes 11 items, comprises three subscales that evaluate civic participation, social cohesion, and reciprocity. A previous study found that there is a correlation between social cohesion and reciprocity (r = 0.4). [31] Civic participation refers to five types of participation in groups: volunteering, sports, hobbies, learning, and experience transfer. Participants were awarded 1 point for each item they participated in at least once a month. Social cohesion refers to trust (“Do you think people in your area are generally trustworthy?”), social reciprocity (willingness to serve others), and attachment to the place of residence, with each item awarded 1 point for a response of “very” or “moderately.” Reciprocity refers to providing and receiving emotional support (worries and complaints) and instrumental support (having someone who provides nursing or care when you are sick in bed for several days or offering someone else that support). If such an individual exists for an item, 1 point is awarded; otherwise, −1 point is awarded. The scores for each answer were summed to obtain the social capital at the individual level, with higher scores indicating a higher degree of social capital. [31] For community-level social capital, percentages of responses for each of the 11 indicators were calculated and aggregated by different voting districts. [31]

#### Other information

Information on the population, number of older adults, and number of LTCI users (percentage of people requiring LTC) was also collected with the cooperation of Uwajima City.

### Statistical analyses

A multilevel logistic regression analysis was performed, in which 65 voting districts were used as stratification variables and the effect of social capital for each stratum considered.

Some divergent regional factors that could potentially affect health outcomes across 76 voting districts needed to be controlled for. In the analysis, differences in the original voting districts could act as confounding factors for differences in the association between social capital and the state of LTC and IADL at the community level. Therefore, we added the aging rate and socioeconomic status (SES; percentage of those with poor economic status and compulsory education in each voting district) to Models 2–4 as regional factors that could affect the results.

Only the social capital of the 65 voting units, which was considered a regional-level factor, was entered into Model 1. In Model 2, we added confounding factors (aging rate and SES) and individual-level social capitals (civic participation, social cohesion, and reciprocity) for the voting interval to Model 1. The reason for adding individual-level social capital was to determine the contextual impacts of community-level social capital on the conditions requiring long-term care and the incidence of IADL disability. In Model 3, we added social environment factors such as gender, age, ES, and education to Model 2. In Model 4, we added the risk factors for LTC need, such as medical history (yes or no), history of falling (yes or no), and sleep (sufficient or insufficient), in that order, to Model 3. The ES was converted from the original four categories to two: rather comfortable and rather uncomfortable. Anyone with a history of hypertension, diabetes, or stroke was considered to have a medical history. Medical histories were chosen as hypertension, diabetes, and stroke as they are clearly documented as the risk factors for LTC. [32]

Model comparisons among models 1–4 are aimed at checking whether community-level social capital is confounded by individual explanatory variables. The odds ratios (ORs)—each social capital parameter divided by the standard deviation—were added to the models; the OR for social capital represents the OR for a one standard deviation increase.

Next, using Model 4, we divided the 65 voting districts into three groups (high, medium, and low-value) according to the tertiles of each subscale score of civic participation, social cohesion, and reciprocity to estimate the proportion of individuals experiencing IADL decline and requiring LTC by using a multilevel logistic regression model (random intercept). Statistical significance was set at less than 5%. P-values for significance levels of 5% and above but below 10% are shown in Tables 2 and 3. SAS version 9.4 (SAS Institute, Cary, NC, USA) was used for the statistical analysis.

## Results

The questionnaires were completed by 6,528 individuals (response rate: 49.4%).

The population characteristics are presented in Table 1. Of the 5,964 individuals evaluated, 217 (3.6%) required LTC, whereas 1,240 (20.8%) reported a decline in IADL. The study population included 2,649 men and 3,315 women, with an average age of 69.4 years (SD = 2.8). Regarding educational background, 51.1% had completed high school, whereas 28.6% had only received compulsory education. Regarding ES, 4.7% of the respondents described themselves as comfortable.

**Table 1:** Population characteristics (n = 5,964)

**Item**	**Values**	
Age (years), n (%)		
65–69	3,143	52.7
70–74	2,786	46.7
Not available	35	0.6
Gender, n (%)		
Men	2,649	44.4
Women	3,315	55.6
Certification of long-term care needs, n (%)		
Not certified	5,732	96.1
Certified (above level 2)	127	2.1
Certified (less than level 2)	90	1.5
Not available	15	0.3
Instrumental activities of daily living (TMIG-IC), n (%)		
≥11 points	4,544	76.2
≤10 points	1,240	20.8
Not available	180	3.0
Educational background, n (%)		
Compulsory education	1,706	28.6
High school, etc.	3,045	51.1
College, etc.	1,117	18.7
Other	63	1.1
Not available	33	0.6
Economic status, n (%)		
Comfortable	281	4.7
Rather comfortable	2,258	37.9
Rather uncomfortable	2,709	45.4
Poor	654	11.0
Not available	62	1.0
Medical history, n (%)		
Yes	2,937	49.2
No	3,026	50.7
Individual-level social capital, mean (SD)		
Civil participation	0.68 (1.07)	
Social cohesion	1.76 (1.13)	
Reciprocity	2.86 (0.50)	
Community-level social capital, mean (SD)		
Civic participation	51.64 (12.17)	
Social cohesion	140.58 (18.18)	
Reciprocity	200.46 (4.67)	

TMIG-IC: Tokyo Metropolitan Institute of Gerontology Index of Competence

SD: standard deviation

Table 2 shows the results of the multilevel logistic regression analysis of the relationship between social capital and the prevention of LTC. Model 1, which introduced social capital only, showed a significant relationship between the prevention of the need for LTC and community-level reciprocity. In Model 2, in which confounding factors (aging rate and SES) and individual-level social capital were added to the precincts, the relationship between the prevention of the conditions requiring long-term care and the reciprocity at the community level weakened temporarily. However, in Model 3, added with individuals’ gender, age, and circumstances (ES), significant relationships were again observed. The OR for this result was 0.85 (95% confidence interval [CI]: 0.74–0.98) per 1 SD increase in reciprocity score. Moreover, after adjusting for risk factors for the conditions requiring long-term care, such as medical history, falls, and sleep (Model 4), higher reciprocity in the community was associated with a low prevalence of the conditions requiring long-term care, although the relationships weakened again (OR, 0.86; 95% CI: 0.75–1.00). Individual-level social capital tended to reduce the need for LTC with increased civic participation and reciprocity.

**Table 2:** ORs and 95% CIs of social capital concerning the need for long-term care

**Variables**	**Model 1**		**Model 2**		**Model 3**		**Model 4**	

	**OR**	**(95% CI)**	**OR**	**(95% CI)**	**OR**	**(95% CI)**	**OR**	**(95% CI)**
Deviation	1219.65		1191.02		1124.48		1030.11	
**Contextual factors**								
Social capital (Community-level):								
Civic participation^a^	1.03	(0.88–1.22)	1.04	(0.87–1.24)	1.00	(0.84–1.19)	1.00	(0.84–1.20)
Social cohesion^a^	0.89	(0.75–1.05)	0.93	(0.76–1.14)	0.91	(0.75–1.11)	0.96	(0.79–1.18)
Reciprocity^a^	0.81	(0.70–0.94)***	0.87	(0.75–1.00)^†^	0.85	(0.74–0.98)*	0.86	(0.75–1.00)^†^
**Compositional factors**								
Social capital (Individual-level):								
Civic participation^a^			0.70	(0.55–0.89)***	0.78	(0.61–0.99)*	0.78	(0.61–1.00)^‡^
Social cohesion^a^			0.91	(0.77–1.07)	0.93	(0.79–1.10)	1.01	(0.84–1.20)
Reciprocity^a^			0.66	(0.53–0.83)***	0.71	(0.56–0.90)**	0.78	(0.59–1.00)^§^
Gender								
Women					1.00		1.00	
Men					1.50	(1.04–2.18)*	1.42	(1.02–2.09)^†^
Age (years)								
65–69					1.00		1.00	
70–74					1.41	(0.98–2.04)^†^	1.26	(0.86–1.84)
Economic status								
Rather comfortable					1.00		1.00	
Rather uncomfortable					1.17	(0.77–1.76)	1.05	(0.69–1.62)
Educational background								
Primary school or less					1.00		1.00	
High school					0.69	(0.46–1.03)^†^	0.76	(0.50–1.16)
College or higher					0.44	(0.23–0.84)*	0.51	(0.27–0.99)*
Others					0.56	(0.08–4.24)	0.57	(0.07–4.50)
Medical history (hypertension, diabetes, stroke, or none of these)								
No							1.00	
Yes							2.37	(1.56–3.60)***
History of falling								
No							1.00	
Yes							3.43	(2.34–5.05)***
Sleep								
Enough							1.00	
Not enough							1.53	(1.02–2.30)*
**Random effects**								
Variance (SE)	0.028(0.078)		0.016 (0.070)		0.013 (0.070)		0.004 (0.075)	

*p < 0.05, **p < 0.01, ***p < 0.001, ^†^p = 0.07, ^‡^p = 0.05, ^§^p = 0.06

Odds ratio were tested in a multilevel logistic regression analysis.

^a^ORs of social capital represent per standard deviation increase of each score.

Variance (SE) in the null model is 0.041 (0.089).

Model 1: Only the social capital of the 65 voting units, which was considered a regional-level factor

Model 2: Model 1 + confounding factors for the voting district (aging rate, percentage of compulsory education, percentage of uncomfortable) + Individual-level social capital (Civic participation, Social cohesion, Reciprocity)

Model 3: Model 2 + Individual-level social environment factors (gender, age, socioeconomic status, education)

Model 4: Model 3 + Individual-level risk factors (medical history, history of falling, sleep)

OR: Odds ratio; CI: Confidence interval; SE: Standard error.

Table 3 shows the results of the multilevel logistic regression analysis regarding the relationship between social capital and decline in IADL. In Model 4, which included all variables entered in the same manner as in Table 2, social cohesion was related to a reduced decline in IADL (OR, 0.87; 95% CI: 0.79–0.96). Individual-level social capital was found to be associated with a reduction of IADL decline in all subscales (civic participation, social cohesion, and reciprocity).

**Table 3:** ORs and 95% CIs of social capital concerning a reduction in instrumental activities of daily living

**Variables**	**Model 1**		**Model 2**		**Model 3**		**Model 4**	

	**OR**	**(95% CI)**	**OR**	**(95% CI)**	**OR**	**(95% CI)**	**OR**	**(95% CI)**
Deviation	5977.59		5219.86		4856.72		4740.08	
**Contextual factors**								
Social capital (Community-level):								
Civic participation^a^	0.99	(0.91-1.06)	1.08	(0.97–1.14)	1.07	(0.98–1.17)	1.07	(0.98–1.17)
Social cohesion^a^	0.87	(0.82-0.93)***	0.90	(0.82–0.98)*	0.86	(0.79–0.94)**	0.87	(0.79–0.96)**
Reciprocity^a^	0.92	(0.86-0.98)*	0.97	(0.90–1.04)	0.95	(0.88–1.03)	0.96	(0.88–1.03)
**Compositional factors**								
Social capital (Individual-level):								
Civic participation^a^			0.47	(0.42–0.53)***	0.53	(0.47–0.59)***	0.53	(0.48–0.59)***
Social cohesion^a^			0.73	(0.69–0.78)***	0.76	(0.71–0.81)***	0.79	(0.74–0.84)***
Reciprocity^a^			0.41	(0.35–0.47)***	0.42	(0.37–0.49)***	0.43	(0.38–0.50)***
Gender								
Women					1.00		1.00	
Men					2.48	(2.15–2.86)***	2.59	(2.23–3.00)***
Age (years)								
65–69					1.00		1.00	
70–74					1.14	(0.99–1.32)^†^	1.14	(0.98–1.31)^‡^
Economic status								
Rather comfortable					1.00		1.00	
Rather uncomfortable					1.69	(1.44–1.98)***	1.60	(1.36–1.88)***
Educational background								
Primary school or less					1.00		1.00	
High school					0.68	(0.58–0.80)***	0.70	(0.60–0.83)***
College or higher					0.58	(0.46–0.73)***	0.60	(0.48–0.76)***
Others					0.60	(0.28–1.28)	0.62	(0.29–1.35)
Medical history (hypertension, diabetes, stroke, or none of these)								
No							1.00	
Yes							1.19	(1.03–1.38)*
History of falling								
No							1.00	
Yes							1.53	(1.27–1.84)***
Sleep								
Enough							1.00	
Not enough							1.63	(1.38–1.91)***
**Random effects**								
Variance (SE)	0.007 (0.021)		0.028(0.022)		0.026 (0.024)		0.036 (0.027)	

*p < 0.05, **p < 0.01, ***p < 0.001, ^†^p = 0.07, ^‡^p = 0.09

Odds ratio were tested in a multilevel logistic regression analysis.

^a^ORs of social capital represent per standard deviation increase of each score.

Variance (SE) in the null model is 0.058 (0.026).

Model 1: Only the social capital of the 65 voting units, which was considered a regional-level factor

Model 2: Model 1 + confounding factors for the voting district (aging rate, percentage of compulsory education, percentage of uncomfortable) + Individual-level social capital (Civic participation, Social cohesion, Reciprocity)

Model 3: Model 2 + Individual-level social environment factors (gender, age, socioeconomic status, education)

Model 4: Model 3 + Individual-level risk factors (medical history, history of falling, sleep)

OR: Odds ratio; CI: Confidence interval; SE: Standard error.

There was no association between community-level civic participation and decreased IADL or the need for LTC.

**Fig. 1:**
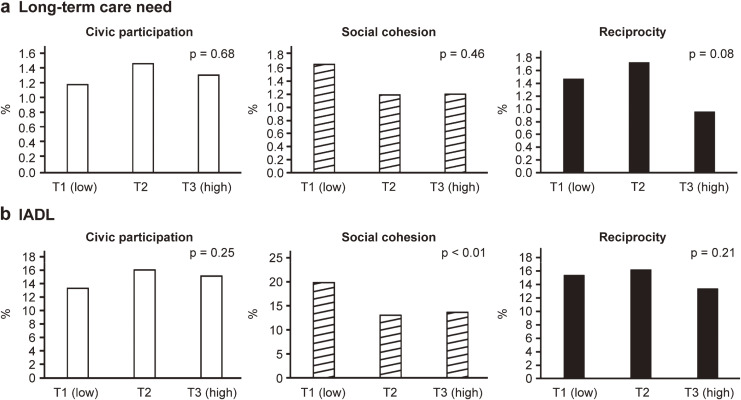
Estimated prevalence of long-term care (a) and decreased IADL (b) by social capital sub-group tertiles.

Figure 1-a shows the subscales of social capital, civic participation, social cohesion, and reciprocity, each of which was divided into three groups by tertiles to estimate the proportion of participants requiring LTC. Among these three subscales, reciprocity tended to be associated with LTC, whereas high reciprocity groups showed a lower prevalence of the LTC than low- and intermediate-reciprocity groups by approximately 0.5–0.7% (p = 0.08). Figure 1-b similarly provides an estimation of the IADL reduction rate; the proportion of people with low IADL decreased by approximately 6% in areas with intermediate and high social cohesion compared to areas with low social cohesion (p < 0.01).

## Discussion

The results revealed that individual-level social capital had a greater impact on LTC need prevention and reduction in the decline in IADL than community-level social capital. There was a tendency for a decline in the need for LTC when individual-level civic participation and reciprocity were high, supporting previous studies. [5, 6] Regarding IADL, a decline was suppressed in all three scales of individual-level social capital. This suggests that individual-level social capital may be useful with regard to LTC need prevention and a reduced decline in IADL. Community-level social capital, on the contrary, can affect everyone in the community.

We used a multilevel model to examine how community-level social capital in daily life was associated with the need for LTC or IADL impairment in older adults. After the addition of several community- and individual-level confounding factors involving the three social capital subscale scores, we found that living in regions with high reciprocity tended to reduce the need for LTC; and living in an environment characterized by high community-level social cohesion was associated with a reduced decline in IADL.

To our knowledge, this is the first study to describe the association between high community-level reciprocity and the need for LTC. Several pathways linking community reciprocity and health have been proposed. [33, 34] Although there has been no direct research on reciprocity and prevention of the need for LTC, previous studies have shown that older adults with five or more friends are more likely than others to be physically active. [35] Further, among older adults, the lack of a strong bond with neighbors and friends has been linked to a lack of protein, calcium, and vitamin intake as well as a lack of physical activity. [36] These previous results do not contradict this study’s findings, wherein living in a highly reciprocal community may reduce the occurrence of LTC needs. Reciprocity refers to the willingness to help neighbors, with the expectation that the favor will be returned in the future; this implies a two-way relationship. [37] There are many theories as to why Japan is among the countries with the highest longevity, but it is well known that one of the reasons is the richness of social capital. [38] It was hypothesized that through this two-way relationship with one’s neighbors, emotional support is accepted and provided, and the richness of this emotional interaction is associated with health.

Furthermore, older adults spend most of their daily lives in the same living sphere, and no other generation depends more heavily on social connections. [12] Therefore, in this study, we determined the community-level social capital based on voting districts, which lie within a 3 km walk from voters’ homes. School districts are usually used as the minimum unit for multilevel analyses, but we consider voting districts to reflect the living sphere of older adults better. School districts in Japan are not uniform in scope, ranging from an easy commuting distance by foot to that by bus, due to the recent consolidation of schools with the declining birth rate. Therefore, social capital is not evaluated in certain small geographical units. While previous studies [5–11, 23, 27, 39–41] used data from multiple municipalities, resulting in different school district areas and unclear distances between living areas, this study is significant in that the areas are clear and was conducted for older adults in one municipality.

Regarding community-level social capital in daily life, it is worth noting that the higher the community-level social cohesion, the lower the IADL disability, and the higher the reciprocity, the lower the need for LTC, even after adjusting for covariates including individual-level social capital variables. However, it is difficult to explain the different roles of reciprocity and social cohesion. In previous assessments of the relationship between social cohesion and psychological and physical roles, higher levels of social cohesion were associated with a decline in depressive sympctoms [42] and frailty. [43] This does not contradict this study’s findings, wherein living in an area of high social cohesion was associated with a reduced decline in IADL. Social cohesion involves trust in others and attachment to the community. [31] A given member of a group may be an uncooperative and mistrusting individual, but they may reside in a community where others are trusting and helpful toward one another. In that case, the uncooperative individual is likely to benefit from the generosity of their neighbors. [13] For example, this person might benefit from being able to shop for daily necessities if there is a neighbor who takes him/her shopping by car, even if the person himself/herself does not have a car. Thus, social activities, such as the purchase of daily necessities and going out, which contribute to older adults’ mental health, increase if they live in areas with a high degree of social cohesion.

Reciprocity, contrastingly, falls under social support, and social support is considered a part of cognitive social capital; [37] the developer of this scale also describes reciprocity as a feature of the region that facilitates the giving and receiving of social support. [31] Previous studies have reported a relationship between social support and cardiac disease and stroke [44] and the incidence of other issues, such as a decline in activities of daily living [45] and smoking. [46] Several studies have reported that people who received more emotional support had a lower risk of early death than those who received less emotional support. [47, 48] However, these were individual-level studies, and the impact of these factors at the community level was unclear. This study’s results suggest that this relationship may hold at both the individual and community levels. Kawachi distinguished social support and social capital as follows: [6] social capital encompasses the health benefits provided by social support in intimate relationships and health impacts, even from weak relationships and acquaintances. In other words, we hypothesized that, in the presence of high community-level reciprocity, even weak relationships with neighbors contribute to lowering the incidence of ADL and stroke—the risk factors that warrant the certification of LTC just for living there. In turn, lower rates of these risk factors would be associated with the prevalence of LTC needs at the individual level. This hypothesis requires further investigation.

Distinguishing civic participation, social cohesion, and reciprocity dimensions is fundamental in social capital studies. [37, 49] As the reciprocity of this scale [31] was correlated with social cohesion, this study’s results show that social cohesion and reciprocity within a community are closely related. Thus, the relationship between the two is complementary, and when either one increases, the other also rises.

Next, higher civic participation at the individual level tended to decrease the need for LTC, but no association was found at the community level. Likewise, civic participation reduced the lowering of IADL at the individual but not the community level. Previous studies suggested that at the community-level, higher civic participation status is protective of health problems among older adults. [5, 6, 50] However, no significant association was found with civic participation. The degree of civic participation differs with living environment and gender. [51–53] We did not observe any impacts of community-level civic participation in this study, which may be due to the absence of a significant difference in the civic participation at individual levels between different voting districts because this survey was conducted in a single local municipality. If a future study were to conduct a survey in voting districts of multiple municipalities, differences in civic participation at the individual level may affect the association with civic participation. Further research is needed to verify whether the association between civic participation, IADL, and the need for LTC can also be observed in familiar voting districts.

### Strengths

Given the rapid population aging, older adults’ care needs are becoming more important every day. Therefore, exploring the preventive role of social capital regarding IADL decline and LTC needs is extremely important. This study has several important policy implications. Given this study’s findings, the promotion of community development with local emotional interaction may reduce the decline in IADL and decrease the rate of certification of the need for nursing care. Specifically, it is important to foster familiar reciprocity within daily living areas of elderly people. It should be two-way relationships, in which elderly people themselves provide support to the people around them as much as possible rather than receiving emotional support one-sidedly. Consequently, it is important to promote the development of communities in which the places are continuously expanded, where elderly people can visit within their familiar communities.

### Limitations

Despite the significance of the findings, several limitations associated with this study warrant mention. First, although depression may be a confounding factor or effect modifier, depression-related questions were not asked. Second, owing to the cross-sectional design, it remains unknown whether current social capital will help reduce future needs for nursing care and decrease social activity competence. Third, while 10% of the surveyed population accounted for nursing care needs in the relevant local government, only 3.6% of the participants required nursing care; thus, this population is not representative of all older adults. Additionally, the response rate was 49.4%, therefore, it is difficult to determine whether the results reflect all the elderly people in Uwajima City. Fourth, the lack of differences in the frequency of civic participation among the participants in this study may be due to the survey being conducted in a single local city. Therefore, to increase generalizability, future studies should accumulate more study results in voting districts of multiple municipalities.

However, given the fixed sample size and the importance of measures to assess social activity competence among older adults who do not require nursing care, these findings will help verify the direction of future care prevention.

## Conclusions

Regarding the relationship between IADL, LTC needs, and social capital, the effect of individual-level social capital factors was larger than that of community-level factors. Regarding community-level social capital, living in a community with high reciprocity levels tended to reduce the need for LTC and living in areas with high social cohesion could reduce the decline in IADL. In Japan, community health workers enthusiastically organize activities to foster community-level social capital. This study’s results provide evidence supporting their efforts and help establish care prevention measures to be implemented in regions where aging is rapidly progressing.

## Abbreviations

CI: confidence interval
ES: economic status
IADL: instrumental activities of daily living
LTC: long-term care
LTCI: long-term care insurance
OR: odds ratio
SES: socioeconomic status

## Supplementary information

**Additional file 1:** Flow chart of the selection of the analyzed participants. Flow chart demonstrating how the participants who were included in the analysis were selected.

**Additional file 2:** Number of participants by voting distinct. The breakdown of the number of participants by voting districts and the number of responses.

## Declarations

### Availability of data and materials

The datasets used and/or analyzed during the current study are available from the corresponding author on reasonable request.
